# Screening and brief interventions for hazardous alcohol use in accident and emergency departments: a randomised controlled trial protocol

**DOI:** 10.1186/1472-6963-9-114

**Published:** 2009-07-03

**Authors:** Simon Coulton, Katherine Perryman, Martin Bland, Paul Cassidy, Mike Crawford, Paolo Deluca, Colin Drummond, Eilish Gilvarry, Christine Godfrey, Nick Heather, Eileen Kaner, Judy Myles, Dorothy Newbury-Birch, Adenekan Oyefeso, Steve Parrott, Tom Phillips, Don Shenker, Jonathan Shepherd

**Affiliations:** 1 Centre for Health Service Studies, University of Kent, Canterbury, UK; 2 Section of alcohol research, Institute of Psychiatry, Kings College, London, UK; 3 Department of Health Sciences, University of York, York, UK; 4 Teams Family Practice, Gateshead, UK; 5 Department of Psychological Medicine, Imperial College, London, UK; 6 Institute of Health and Society, Newcastle University, Newcastle, UK; 7 Northern Regional Drug and Alcohol Services, Newcastle, UK; 8 School of Psychology and Sports Science, University of Northumbria, UK; 9 Division of Mental Health, St George's University of London, UK; 10 Humber Mental Health and Teaching NHS Trust, Willerby, UK; 11 Alcohol Concern, London, UK; 12 Violence Research Group, Cardiff University, Cardiff, UK

## Abstract

**Background:**

There is a wealth of evidence regarding the detrimental impact of excessive alcohol consumption on the physical, psychological and social health of the population. There also exists a substantial evidence base for the efficacy of brief interventions aimed at reducing alcohol consumption across a range of healthcare settings. Primary research conducted in emergency departments has reinforced the current evidence regarding the potential effectiveness and cost-effectiveness. Within this body of evidence there is marked variation in the intensity of brief intervention delivered, from very minimal interventions to more intensive behavioural or lifestyle counselling approaches. Further the majority of primary research has been conducted in single centre and there is little evidence of the wider issues of generalisability and implementation of brief interventions across emergency departments.

**Methods/design:**

The study design is a prospective pragmatic factorial cluster randomised controlled trial. Individual Emergency Departments (ED) (n = 9) are randomised with equal probability to a combination of screening tool (M-SASQ vs FAST vs SIPS-PAT) and an intervention (Minimal intervention vs Brief advice vs Brief lifestyle counselling). The primary hypothesis is that brief lifestyle counselling delivered by an Alcohol Health Worker (AHW) is more effective than Brief Advice or a minimal intervention delivered by ED staff. Secondary hypotheses address whether short screening instruments are more acceptable and as efficient as longer screening instruments and the cost-effectiveness of screening and brief interventions in ED. Individual participants will be followed up at 6 and 12 months after consent. The primary outcome measure is performance using a gold-standard screening test (AUDIT). Secondary outcomes include; quantity and frequency of alcohol consumed, alcohol-related problems, motivation to change, health related quality of life and service utilisation.

**Discussion:**

This paper presents a protocol for a large multi-centre pragmatic factorial cluster randomised trial to evaluate the effectiveness and cost-effectiveness of screening and brief interventions for hazardous alcohol users attending emergency departments.

**Trial Registration:**

ISRCTN 93681536

## Background

### Prevalence of alcohol use disorders

The recent Alcohol Needs Assessment Research Project for England (ANARP) reported that 26% (38% of men and 16% of women) of the population of England aged 16–64 have an alcohol use disorder (AUD), equivalent to approximately 8.2 million people [1]. Alcohol use disorders include hazardous drinking, harmful drinking and alcohol dependence. The prevalence of alcohol dependence overall was 3.6%, with 6% of men and 2% of women meeting these criteria nationally, which equates to 1.1 million people. The rising patterns in consumption suggest that the prevalence of AUD will increase significantly over the next ten years, which poses a significant threat to the health of the UK population.

Over 14 million people are treated in Emergency Departments (ED) in England each year, of which 35% of attendances are alcohol related at a cost of over £0.5 billion per year [2]. Furthermore, a recent survey of 32 EDs in England found that up to 40% of admissions at weekends and up to 70% at peak times were alcohol related [2]. The high levels of attendances can be explained by the link between excessive drinking and a greater risk of being involved in accidents, assaults, fights and other traumatic events requiring hospital care [3-5]. The vast number of patients that visit ED each year with or without alcohol related presentations offers the opportunity to access and intervene with a large number of patients who may misuse alcohol.

### Current evidence for screening and brief interventions

A wide array of screening methods has been developed including verbal instruments or questionnaires. Examples of paper based questionnaires include the Alcohol Use Disorders Identification Test, AUDIT [6], Fast Alcohol Screening Test, FAST [7], Michigan Alcoholism Screening Test [8] and the Paddington Alcohol Test, PAT [9]. All have an acceptable, and in some cases a remarkably high, level of sensitivity and specificity compared to more complex research measures of excessive drinking and alcohol use disorders. For example a recent UK primary care study using the AUDIT found it to have 92% sensitivity and 92% specificity for hazardous and harmful drinking compared with a Time Line Follow Back (gold standard) measure of alcohol consumption [10]. Questionnaire methods of screening are considerably more valid and cost effective than blood screening methods [10]. However, the various methods differ in several respects: CAGE and FAST are 4 item measures; AUDIT is 10; the PAT is designed to be incorporated into routine ED assessment, whereas the FAST has been tested primarily in a research context of universal screening. This has led to the development of shorter variants of questionnaires for use in busy clinical settings (e.g. FAST and AUDIT C are shorter versions of AUDIT). Indeed some US studies showed that a single item measure of excessive drinking (SASQ) had 86% sensitivity and specificity for alcohol use disorders [11,12]. Further, recent pilot work using focus groups in primary care showed that healthcare practitioners were more likely to adopt shorter instruments, that were simpler to score and that were less disruptive to clinical practice. The poorer screening performance of shorter instruments of lower sensitivity may be offset in the typical busy clinical situation by greater use by practitioners. Further, questionnaire items that enquire simply about alcohol consumption, particularly when embedded in a general health and lifestyle questionnaire, are more likely to be acceptable to clinicians and patients as being less stigmatizing.

Similarly, whilst much of the research on alcohol screening methods has been carried out in a universal screening paradigm there is evidence to suggest that targeted screening, restricted to higher risk individuals, is more acceptable to practitioners [13]. Indeed PAT has been designed to be used among patients with one of 10 common alcohol related conditions presenting to ED and so functions as a targeted screening approach. Pre-screens involving enquiry about medical conditions associated with alcohol use are potentially more acceptable to both patients and clinicians. However, it is currently unclear which approach, universal or targeted screening, is more effective and cost-effective in the ED setting, hence the need to evaluate this issue in our trial.

There exists a substantial evidence base for the efficacy of brief interventions aimed at reducing alcohol consumption across a range of healthcare settings [14]. A review focusing upon the delivery of brief interventions in accident and emergency departments [15] concluded that the evidence was such that *"...screening and brief intervention for alcohol-related problems in the Emergency Department be incorporated into clinical practice" *(p627). Primary research conducted in accident and emergency departments has reinforced the current evidence regarding the potential effectiveness [16-19] and cost-effectiveness [20,21]. Within this body of evidence there is marked variation in the intensity of brief intervention delivered, from very minimal interventions to more intensive behavioural or lifestyle counselling approaches. Further the majority of primary research has been conducted in single centre and there is little evidence of the wider issues on generalisability and implementation of brief interventions across accident and emergency departments.

### Public health policy context

This research is designed to support the National Alcohol Harm Reduction Strategy for England which has called for "more information...on the most effective methods of targeted screening and brief interventions, and whether the successes shown in research studies can be replicated within the health system in England" [22]. This project aims to address these issues to provide evidence on the delivery, effectiveness and cost effectiveness of a range of alcohol screening and brief intervention approaches across settings and regions in England. It is envisaged that the results of the study will have implications for the implementation and delivery of screening and brief interventions in an international context.

### Aims of the study

The aim of this study is to evaluate the implementation, effectiveness and cost effectiveness of brief intervention to reduce excessive drinking in hazardous and harmful drinkers identified by universal or targeted screening delivered by an Alcohol Health Worker (AHW) in routine ED care compared to brief advice and a patient information leaflet (PIL) delivered by ED staff.

The objectives of the trial are:

• To conduct a pragmatic multicentre cluster randomised controlled trial of screening and brief interventions for hazardous and harmful drinkers in EDs in three English regions.

• To compare the effectiveness and cost effectiveness of brief advice given by ED staff with referral to an AHW of patients with hazardous and harmful alcohol consumption identified by targeted opportunistic or universal screening.

• To assess the relative impact of the three implementation strategies on alcohol screening and brief intervention activity in EDs.

• To identify the attitudinal, practical, skill, resource and reinforcing factors that act as prognostic indicators of successful implementation of screening and brief intervention in ED.

• To identify the optimal method of alcohol screening in ED.

• To assess the relative impact of the three implementation strategies on uptake of alcohol services, including an alcohol helpline.

## Methods/design

**Figure 1:**
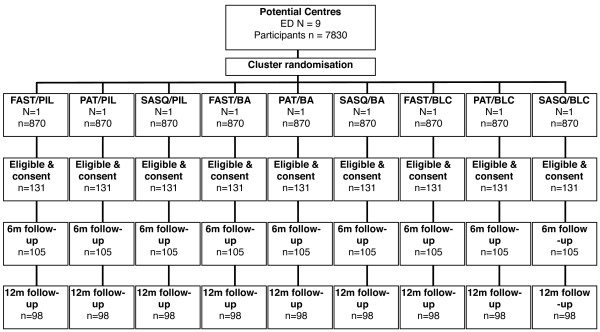
**Trial Flow Chart**.

The study is a pragmatic factorial cluster randomised trial. The study has been granted ethical approval by the London Research Ethics Committee ref: 07/MRE02/06. The study complies with the Helsinki declaration. A full flow diagram for the study is shown in Figure 1.

A number of methodological considerations have been taken into account in designing this study. First, we are conducting in the main a pragmatic rather than an explanatory trial because our focus is primarily upon decision making rather than understanding. Second, we are conducting an effectiveness trial rather than an efficacy trial; our aim is to maximise generalisability of our findings to usual clinical practice and we therefore have maximised external validity. Third, we are concerned about the potential of contamination within ED in terms of both the screening mechanism and the intervention and therefore we have chosen to use cluster randomisation with the ED as the unit of randomisation. Fourth, we aim to evaluate three screening tools (M-SASQ vs FAST vs SIPS-PAT) and three levels of intervention (Minimal intervention vs Brief advice vs Brief lifestyle counselling). This 3 × 3 factorial design allows both screening method and intervention to be analysed separately with sufficient statistical power and for the analysis of any potential interactions between screening method and intervention.

### Hypotheses

• Brief lifestyle counselling (BLC) by an AHW for hazardous and harmful drinkers identified by screening is more effective and cost effective than brief advice (BA) conducted by ED staff in the typical ED setting.

• Brief lifestyle counselling by an AHW and brief advice by ED staff are more effective and cost effective than an information leaflet alone (PIL).

• Deployment of an AHW results in greater screening and intervention activity than training of ED staff in screening and brief intervention alone.

• Briefer screening methods result in greater implementation of screening activity than more complex methods.

### Trial inclusion and exclusion criteria

Inclusion and exclusion criteria have been chosen to maintain a balance between ensuring the sample is representative of the ED population whilst ensuring that the trial population is able to engage both with the interventions and follow up.

All ED attendees aged 18 or more who score positive for AUD, who are alert and orientated, live within the local area of the ED and are able to speak, read and write English sufficiently well to complete study questionnaires are eligible. Patients are not eligible if they are already seeking treatment for an AUD or are taking part in another study of alcohol interventions, if they are severely injured or suffering from a serious mental health problem and/or are grossly intoxicated. Finally patients with no fixed abode are excluded from the study.

### Randomisation

Randomisation will be conducted using a secure remote randomisation service. Nine allocations will be generated for each of the possible factorial combinations of screening method (SIPS-PAT vs FAST vs M-SASQ) and intervention (PIL vs BA vs BLC). EDs and allocations will be randomly sampled without replacement and paired to generate allocation groups. The allocations are detailed in Table 1.

**Table 1:** 3 × 3 allocation table

		**Intervention allocation**			
		PIL	BA	BLC	
**Screening allocation**	FAST	AED = 1	AED = 1	AED = 1	**AED = 3**
		n = 131	n = 131	n = 131	**n = 393**
	SASQ	AED = 1	AED = 1	AED = 1	**AED = 3**
		n = 131	n = 131	n = 131	**n = 393**
	PAT	AED = 1	AED = 1	AED = 1	**AED = 3**
		n = 131	n = 131	n = 131	**n = 393**
		**AED = 3**	**AED = 3**	**AED = 3**	**AED = 9**
		**n = 393**	**n = 393**	**n = 393**	**n = 1179**

### Screening

In order to test the relative effectiveness of different screening methods in identifying hazardous and harmful drinkers we intend to conduct a cluster randomized comparison of three established screening tools. A modified version of the Paddington Alcohol Test (PAT) [9] which has been routinely used at St. Mary's Hospital Paddington embedded within the standard clinical assessment process. The modified PAT (SIPS-PAT) retains the top 10 screening conditions but has been shortened to 2 questions. Question 1 asks "Do you feel your attendance here is related to your drinking?" if the answer is Yes then the patient is considered SIPS-PAT positive; if the answer is No, a further question is asked, "How often do you have X or more standard drinks on one occasion?" where X = 6 for women and 8 for men, with monthly, weekly or daily considered a positive screen. The original PAT has undergone validity testing in ED as a targeted screening tool and has been found to have high sensitivity and specificity for identifying AUD in this population [9]. A modified version of the Single Alcohol Screening Questionnaire (M-SASQ) which asks "How often do you have X or more standard drinks on one occasion?" where X = 6 for women and 8 for men, with monthly, or weekly, or daily or almost daily considered a positive screen. The original SASQ has undergone validity testing in primary care settings and has a high level of sensitivity and specificity for alcohol use disorders, although lower than PAT [11,12]. SASQ is shorter than PAT and while it has lower sensitivity and specificity it may be more acceptable to ED staff. We tested the M-SASQ in a pilot study and established a sensitivity of 91.8 and specificity of 70.8 when compared to the gold standard Alcohol Use Disorders Identification Test (AUDIT). The Fast Alcohol Screening Test (FAST) [7] is a universal screening tool that has demonstrated high sensitivity and specificity in ED settings. All screening tools have been modified to include a visual guide to interpreting a 'standard drink' which equates to 8 mg of pure ethanol.

In accordance with best practice all patients who are attending ED and potentially eligible will be informed of the study and asked to consent to be screened. They will be screened using the instrument available within the ED. Participants will be informed of the outcome of the screening.

### Consent

Consent to participate will be obtained in a 2-stage process. ED staff will initially establish verbal consent to check eligibility to take part, collect some basic demographic information and consent to be screened. Those who then are positive on the screening tool will have the study explained to them verbally by ED staff and in writing (using the patient information sheet). Written informed consent will be obtained by ED staff. This will include permission to give the patient's data and contact details to the research staff, provide the research team with access to the patients ED records and to participate in follow up after 6 and 12 months. The research team will then contact the patient within two weeks to thank him/her for taking part in the study. Participants will receive a £10 retail voucher after completing the baseline research interview and a £10 voucher for completing each of the 6 and 12 month research follow-up interview.

#### Patient information leaflet

In the three EDs randomised to the control condition, participating staff will be trained to screen eligible patients for hazardous or harmful drinking and patients will not have access to a AHW. Patients who screen positive and provide consent to participate in the study will complete the baseline questionnaire and then be provided with a patient information leaflet (PIL) . The PIL to be used in this trial will be the Department of Health's "How much is too much; Drinking and You" leaflet. This information leaflet contains useful information about alcohol and includes the Drinkline telephone number where the patient can access further information including treatment options for alcohol problems. A sticker with local alcohol services will also be attached to the back cover.

### Brief advice

In the three EDs randomized to deliver Brief Advice, participating staff will be trained to screen eligible patients for hazardous or harmful drinking. Patients who screen positive and provide consent to participate in the study will complete the baseline questionnaire and will then receive up to five minutes of simple structured brief advice from trained ED staff or an AHW, using the SIPS Brief Advice tool "Brief advice about alcohol risk" which has been developed for the SIPS programme. It is based on the "How much is too much? Simple Structured Advice intervention tool, developed as part of the UK version of the Drink-Less BI programme [23] from a prototype used as part of a World Health Organisation collaborative study on alcohol screening and brief. Patients in this condition will also receive a Patient Information Leaflet (PIL) at the end of the brief advice.

### Brief lifestyle counselling

In the three EDs randomized to deliver Brief Lifestyle Counseling, participating staff will be trained to screen eligible patients for hazardous or harmful drinking. Patients who screen positive and provide consent to participate in the study will complete the baseline questionnaire and will then receive up to five minutes of simple structured brief advice from trained ED staff, using the SIPS Brief Advice tool "Brief advice about alcohol risk". Patients in this condition will also receive a Patient Information Leaflet (PIL) at the end of the brief advice. ED staff will also be trained to refer patients to an AHW by making an appointment usually the following day or as soon as possible after ED attendance. The AHW will be experienced in carrying out alcohol assessment and brief interventions. The AHW will deliver a 20 minute brief lifestyle counseling intervention to patients who attend the appointment at the ED, using the SIPS Brief Lifestyle Counselling (BLC) Tool which has been developed for the SIPS programme. It is based on the "How much is too much?" Extended Brief Intervention tool developed as part of the UK version of the Drink-Less BI programme [23] from a prototype used as part of a World Health Organisation collaborative study on alcohol screening and brief intervention.

All intervention tools and protocols are available from the SIPS study website .

### Training of staff in trial protocols and screening for AUDs

All ED staff participating in the trial will be trained to implement alcohol screening and brief intervention according to the trial protocol. The aim of the training is to provide some background information about alcohol related harm, to give an overview of the study protocol, to familiarise staff with the screening tools, structure and scoring procedures, and to inform staff about the procedure for implementing screening and brief intervention at their site. Given the cluster design of the trial, staff will only be introduced to the screening tool they have been randomly allocated to. The training is individualised according to the implementation procedure agreed with the local collaborator and senior colleagues in the ED.

A substantial element of the training involves the understanding of and familiarisation with alcohol units to ensure that the practitioners are fully aware of the alcohol content of different alcoholic drinks so they are able to complete the screening tools accurately. Moreover, as the screening tools refer to standard drinks rather than units when assessing consumption, the training ensures staff are aware that a standard drink is one unit and that they are able to convert different drinks into the number of standard drinks e.g. one pint of premium lager equals three standard drinks (or three units). Visual representations of standard drinks as well as several examples of people's drinking patterns are used to allow trainees to practice calculating units in each drink and to add up the number of standard drinks/alcohol units consumed in order to identify positive cases.

### Training of staff to deliver brief advice

Participating staff selected to deliver brief advice in EDs randomised to either the Brief Advice (BA) condition or the Brief Lifestyle Condition (BLC) will receive a one hour training session on how to deliver five minutes of brief advice according to the protocol.

The aim of the training is to provide practitioners with the skills necessary to effectively deliver brief advice about alcohol risk to patients attending the ED in which they work. The training was developed by the SIPS team to be delivered by an AHW. The AHWs in the SIPS team are experienced practitioners in the field of alcohol treatment. They contributed to the development of the training package and have been fully trained to deliver the training to practitioners.

The standard training package is based on a PowerPoint presentation with scripts to standardise delivery. The training sessions are adapted for use in the different experimental conditions in which BA is being delivered.

The session will be presented to small groups of clinicians who are encouraged to interact with the trainer, ask questions and comment on the content. This is followed by an interactive role play session in which the AHW demonstrates the intervention and each practitioner then has an opportunity to practice with a co-worker, observed by the trainer who provides feedback and encouragement. Training sessions are delivered to groups of 1–10 practitioners with 3–4 being the typical group size. However, some EDs require larger groups due to the higher number of participating staff.

### Training of staff to deliver brief lifestyle counselling

AHWs will be recruited to deliver the BLC within the randomised EDs. Staff recruited will, as a minimum, possess; a relevant professional qualification, a diploma in drug and/or alcohol studies or equivalent, 5 years post-qualifying experience with a minimum 2 years in an alcohol or drug speciality, prior knowledge and understanding of psychological interventions including motivational interviewing.

All AHWs will receive formal training and supervision from the point of recruitment. Training will be based upon the previous work of Rollnick et al [24] in addition to experiences from an earlier trial of screening and brief interventions [25]. The training will comprise four main elements: orientation to the relevant ED, taught workshops, tape recorded simulated consultations with trained actors and ongoing clinical supervision provided by experienced senior clinicians.

The simulated consultations will be recorded and rated by three independent clinical assessors. The AHW will be assessed for adherence to the BLC protocol in addition to their behaviour and skills using the Behaviour Change Counselling Index (BECCI) [26]. Assessors will submit BECCI ratings, comments and supervision points for each consultation. This information will support clinical supervision and training until the AHW reaches a required standard of practice agreed by an independent clinical assessor.

### Primary outcome measure

The primary outcome measure for the study is the score on the AUDIT screen at 12 months post-consent. This screen is ascertained using the Extended AUDIT [27] a modified form of the AUDIT that exhibits similar sensitivity and specificity to AUDIT.

### Secondary outcome measures

1. Average drinks, where a drink equates to 8 mg of pure ethanol per day at baseline and 6 and 12 months will be established using the quantity-frequency questions of the Extended AUDIT. 2. Health utility will be measured at baseline, 6 and 12 months using the EQ-5D [28]. 3. Service Use Questionnaire will be used to measure service utilisation at baseline, 6 and 12 months. 4. An adapted readiness to change ruler validated for use in this study [29] measured at baseline, 6 and 12 months. 5. Alcohol related problems will be assessed using the Alcohol Problems Questionnaire at 6 and 12 months [30]. 6. Patient satisfaction with the advice received during the intervention will be assessed using a modified version of the Patient Satisfaction Questionnaire (short form) at 12 months [31].

### Economic outcome measures

Screening costs will be estimated using the actual costs of screening using the actual costs of screening associated with the study. Costs of delivering the interventions will be based upon actual patient contact time from time sheets maintained by staff. The units of services used will be based upon local costs of services and include allowances for managerial and premises overheads and the costs associated with training and supervision using methods utilised in similar intervention studies [32]. The costs of any specialist referral will be ascertained using information on the actual costs associated with specialist service provision based upon Department of Health costs of specialist interventions [33]. Participant use of health services, other alcohol services outside the study, public services and criminal justice services will be assessed using a Service Use Questionnaire at baseline, 6 and 12 months post consent. This has been developed over a number of alcohol intervention studies and will be adapted to capture costs specifically associated with this population.

### Quality assurance and process measures

Attendance for brief lifestyle advice on the part of the client will be recorded by the AHW. During the course of the study AHW will receive continuing supervision from senior members of the clinical team replicating ongoing quality assurance in clinical practice. At the end of the trial all AHWs who delivered brief lifestyle counseling will participate in simulated consultation and these in turn will be rated by three independent raters using BECCI. This information will be utilized to establish both adherence to the BLC protocol and as a measure of quality assurance in the analysis.

### Sample size calculation

The sample size calculation is designed to account primarily for intervention level outcomes. Powering the study in this way will also account statistically for appropriate outcomes for screening approach and screening method. The primary outcome for this study is the proportion of patients who consume alcohol within recommended levels at 12 month follow up. Recent meta-analysis [14] suggests that the difference between brief intervention and control is of the order 13%, 5% reduction in the control group and 18% in the brief intervention group. In order to detect a difference of this magnitude at the 5% significance level with 80% power, for a 2-sided test, requires 109 patients in each of the 3 groups, a total of 327. Our experience with other multi-centre randomized controlled trials of interventions for alcohol use disorders suggests that with assiduous follow-up the potential loss to follow-up across groups is of the order 25%. Taking this loss into accounts inflates the sample required to 131 in each group, a total of 393 patients.

The proposed study involves a cluster design and requires a statistical adjustment to account for any potential cluster effect. The literature and our previous experience of trials in primary care suggest that an intra-class correlation coefficient of 0.04 is appropriate. Assuming a cluster size of the order 44 patients, this inflates the sample size calculation by a factor of 2.7 requiring a total of 1179 patients, 393 in each group, with an expectation that at least 882 will be followed up at 6 months and 12 months. We propose to recruit 9 EDs. ED populations will be screened using 1 of 3 screening method groups (3 EDs in each group) and will receive 1 of 3 interventions (3 EDs in each group). Our estimates using data from previous studies suggest that a conservative estimate of 15% of those approached will be eligible and consent. In order to identify 131 eligible and consenting participants, 870 participants will be approached in each of the EDs a total of 7830.

### Effectiveness analysis

As the study is pragmatic in design, the planned analysis will be by intention to treat. The primary outcome is dichotomous in nature, drinking within or above recommended levels, and will be analysed through a logistic regression adjusting for all known prognostic factors. Data will be presented as odds ratios and their corresponding 95% confidence intervals. Secondary analyses will be undertaken using the appropriate method for the outcomes, controlling where appropriate for intake values and other known prognostic variables using analysis of covariance. We will assess the sensitivity and specificity of each screening approach by generation of receiver operator curves and comparison with the gold standard AUDIT screening tool. Simulations will be undertaken to encompass both screening approach groups and intention to treat analysis will be undertaken on both groups. Due to the nested factorial nature of the study, we will use multi-level modelling to explore potential interactions between each of the levels nested within the trial.

ED and patient factors will be utilised as part of regression model to explore possible prognostic factors that impact on outcome. Interaction analysis will explore any possible interactions between ED and patient characteristics and outcome. The efficacy of the interventions will be explored with a secondary analysis utilising a per protocol approach. A sub-group of the trial population, those who engaged in the allocated treatment, will be utilised for this analysis.

### Cost-effectiveness analysis

The economic component of the study comprises a cost-effectiveness and cost-utility analysis. The study aims to identify, quantify and value resources related to alcohol screening and intervention by clinicians in ED and the subsequent use of health, social care and criminal justice services by patients following each type of intervention. Resources utilised in the identification and brief intervention delivery or control condition will be recorded by ED staff involved on an ongoing basis. This will allow the calculation of costs related to implementation of different models of screening and brief intervention. Local costs will be used to calculate the costs of the interventions, which will include staff costs, premises costs and costs of leaflets and other consumables. In addition, specific training costs for staff will be calculated in terms of staff time, premises costs and the cost of training materials.

Patients' use of health, social care and criminal justice services will be identified retrospectively using a short form of the Service Use Questionnaire previously used to evaluate costs associated with interventions for alcohol use disorders and applying a common set of national unit cost estimates. The economic analysis will calculate the incremental cost-effectiveness of the control condition with the AHW condition under study, using measures of clinical outcome and quality of life, EQ-5D responses at baseline, 6 and 12 month follow up. The use of EQ-5D enables the estimation of Quality Adjusted Life Years. Data will be bootstrapped to account for the expected skewness evident in economic cost data [34]. The analysis will include the construction of cost-effectiveness acceptability curves to illustrate the probability that the brief intervention is more cost-effective than usual care, based on different monetary values being attached to QALYs. The use of QALYs follows the recommendations of NICE and enables the value for money afforded by treatment to be compared to a range of other health care interventions. Furthermore, combination of the economic cost data and outcome data with patient data collected in the trial will enable a secondary analysis of various patient characteristics that may influence the cost-effectiveness of the intervention.

### Frequency of analysis

Analysis will be conducted after the final 12 month follow-up has been completed.

### Ethics and confidentiality

The study has been granted ethical approval by a multi-centres research ethics committee and by the local research ethics committee for the localities where the research will take place. There are no anticipated risks in relation to either treatment. There is no documented evidence of adverse events arising from any of the proposed interventions.

All trial data will be identified using a unique trial identification number. No personally identifiable information will be held beyond the final 12 month follow up. Analytical datasets will not contain any patient identifiable information. Anonymised data will be retained for a period of 60 months.

## Discussion

This study is part of programme of research being conducted in England to evaluate the implementation, effectiveness and cost-effectiveness of a variety of approaches to screening and interventions for hazardous and harmful alcohol users across emergency departments, primary health care and criminal justice settings.

## Competing interests

The authors declare that they have no competing interests.

## Authors' contributions

All of the authors contributed to the design and development of the trial protocol. SC and KP wrote the first draft of the paper. SC, KP, MB, PC, MC, PD, CDr, EG, CG, NH, EK, JM, DNB, AO, SP, TP, DS & JS commented and contributed to successive drafts. All authors read and approved the final manuscript.

## Pre-publication history

The pre-publication history for this paper can be accessed here:


